# Bacteremia in Childhood Life-Threatening Infections in Urban Gambia: EUCLIDS in West Africa

**DOI:** 10.1093/ofid/ofz332

**Published:** 2019-07-27

**Authors:** F Secka, J A Herberg, I Sarr, S Darboe, G Sey, M Saidykhan, M Wathuo, M Kaforou, M Antonio, A Roca, S M A Zaman, M Cebey-López, N P Boeddha, S Paulus, D S Kohlfürst, M Emonts, W Zenz, E D Carrol, R de Groot, L Schlapbach, F Martinon-Torres, K Bojang, M Levin, M van der Flier, S T Anderson

**Affiliations:** 1 Medical Research Council The Gambia at London School of Hygiene & Tropical Medicine, United Kingdom; 2 Imperial College London, Section of Paediatric Infectious Disease, United Kingdom; 3 Instituto de Investigación Sanitaria de Santiago, Genetics-Vaccines-Infectious Diseases and Paediatrics Research Group, GENVIP, Spain; 4 Erasmus MC-Sophia Children’s Hospital, University Medical Center Rotterdam, Intensive Care and Department of Paediatric Surgery, The Netherlands; 5 University of Liverpool Institute of Infection and Global Health, Department of Clinical Infection Microbiology and Immunology, United Kingdom; 6 Medical University of Graz, Department of General Paediatrics, Austria; 7 Institute of Cellular Medicine, Newcastle University, Newcastle upon Tyne, United Kingdom; 8 Paediatric Infectious Diseases and Immunology Department, Newcastle upon Tyne Hospitals Foundation Trust, Great North Children’s Hospital, United Kingdom; 10 Paediatric Infectious Diseases and Immunology, Amalia Children’s Hospital, and Expertise Center for Immunodeficiency and Autoinflammation, and Section Paediatric Infectious Diseases, Laboratory of Medical Immunology, Radboud Institute for Molecular Life Sciences, and Radboud Center for Infectious Diseases, Radboudumc, Nijmegen, the Netherlands; 11 University Children’s Hospital Zurich and the Children’s Research Center, Switzerland

**Keywords:** antibiotic, bacteremia, children, Gambia, mortality

## Abstract

**Background:**

The limited availability of microbiology services in sub-Saharan Africa impedes accurate diagnosis of bacterial pathogens and understanding of trends in prevalence and antibiotic sensitivities. We aimed to characterize bacteremia among hospitalized children in The Gambia and to identify factors associated with bacteremia and mortality.

**Methods:**

We prospectively studied children presenting with suspected severe infection to 2 urban hospitals in The Gambia, between January 2013 and September 2015. Demographic and anthropometric data, clinical features, management, and blood culture results were documented. Urine screens for antibiotic activity were performed in a subset of participants.

**Results:**

Of 411 children enrolled (median age, 29 months; interquartile range, 11–82), 79.5% (325 of 409) reported prehospital antibiotic use. Antimicrobial activity by urinary screen for antibiotic activity was detected in 70.8% (n = 80 of 113). Sixty-six bacterial pathogens were identified in 65 (15.8%) participants and *Staphylococcus aureus* predominated. Gram-positive organisms were more commonly identified than Gram-negative (*P* < .01). Antibiotic resistance against first-line antimicrobials (ampicillin and gentamicin) was common among Gram-negative bacteria (39%; range, 25%–100%). Factors significantly associated with bacteremia included the following: gender, hydration status, musculoskeletal examination findings, admission to the Medical Research Council The Gambia at London School of Hygiene & Tropical Medicine hospital, and meeting sepsis criteria. Those associated with increased mortality were presence of a comorbidity, clinical pallor, tachypnea, and altered consciousness. Tachycardia was associated with reduced mortality.

**Conclusions:**

The bacteremia rate in children with suspected childhood life-threatening infectious diseases in The Gambia is high. The pattern of pathogen prevalence and antimicrobial resistance has changed over time compared with previous studies illustrating the importance of robust bacterial surveillance programs in resource-limited settings.

Child mortality in sub-Saharan Africa (SSA) is high, and the majority of these deaths can be attributed to bacterial infections [1–3]. Passive surveillance systems to monitor bacterial infections and associated resistance patterns are not common practice [4], and there are few robust estimates of the burden of bacteremia in children in resource-limited settings [5] due to limited access to microbiology facilities and molecular diagnostic tools [6]. In addition, uncontrolled (prehospital) use of antibiotics in SSA [7] limits reliable identification of bacteria via traditional blood culture. This results in a paucity of reliable data on the prevalence and sensitivity pattern of commonly isolated pathogens over time [2, 8, 9]. Differences in the etiology of pediatric bacterial infections between different geographic regions additionally reduce the efficacy of treatment recommendations and immunization programs in SSA [10–15].

A systematic review in 2010 on community-acquired bacteremia in Africa, which included a study from The Gambia [16], found that *Streptococcus pneumoniae* and *Haemophilus influenzae* were the most common pathogens in the pediatric population. Although predictors of bacteremia have been widely studied in high-income countries [17], these findings cannot be translated to the SSA setting because of epidemiological differences. Studies on predictors of pediatric bacteremia in SSA are limited. A publication from South Africa [18] identified malnutrition to be more common in bacteremic patients, whereas another from Nigeria [19] highlighted 2 other predictors: age less than 6 months and leukocytosis (>15 000 cells/µL). Pulmonary crackles and leukocytosis (>15 000 cells/µL) were risk factors identified in Uganda [20]. Another study did not identify predictors [21]. There are no recent published data on pediatric bacteremia from The Gambia. The last study, which was completed more than 10 years ago and combining all age groups, showed that patients with leukocytosis were more likely to be bacteremic [22].

Studies of community onset sepsis and blood stream infections in SSA in children outside the neonatal period have shown case-fatality rates ranging from 5.2% to 16.6% [9, 13, 18, 23] in children with bacteremia and significant levels of sequelae; one study in West African children under 5 years old reported disability at discharge in 13.2% with confirmed bacteremia [13].

In this study, we investigated the prevalence of bacteremia and describe antibiotic resistance patterns in children with sepsis and severe focal infection (SFI) and the factors associated with bacteremia and mortality.

## MATERIALS AND METHODS

From January 2013 to September 2015, we conducted a prospective, observational study recruiting children presenting with suspected sepsis or SFI to 2 hospitals in urban Gambia. This study was nested within the European Union Childhood Life-threatening Infectious Disease Study ([EUCLIDS] www.euclids-project.eu). The European cohort has been described previously [24].

### Study Participants

Study participants were aged 1 month to less than 18 years with suspected sepsis or SFI, who warranted admission for effective antibiotic treatment. Recruitment was not comprehensive and was limited to working hours by the availability of the research team. Patients referred to the tertiary hospital were not recruited if they were already on intravenous antibiotics or had negative blood cultures at screening.

### Definition of Terms

Sepsis was defined as suspected infection plus systemic inflammatory response syndrome, as per clinical criteria established by international consensus definition [25]. Children not meeting sepsis criteria but with a clinical focus of significant infection were defined as having SFI with a matching syndrome recorded according to the predefined case definitions in the EUCLIDS clinical protocol [24]. In cases wherein limitations arose due to lack of diagnostic investigations, clinical syndromes were defined using criteria detailed in the World Health Organization (WHO) Pocket Book of Hospital Care for Children [26]. Anthropometry was categorized according to standard WHO criteria [27]. Sequelae at discharge were recorded.

### Patient Recruitment

Patients were recruited from 2 hospital settings: the Medical Research Council The Gambia at London School of Hygiene & Tropical Medicine (MRCG at LSHTM) clinical services hospital and the Edward Francis Small Teaching Hospital (EFSTH). Case report forms captured data on sociodemographic details, anthropometry, medical history, clinical features, management, and results of blood culture and antibiotic sensitivity profiles. Data were subsequently transferred to an online, secure study database.

The MRCG at LSHTM, located in the greater Banjul area, runs a 42-bed inpatient unit providing both pediatric and general medical care for staff, the general public, and participants in MRCG at LSHTM-led clinical research studies. It is supported by Good Clinical Laboratory Practice (GCLP) and International Organization for Standardization (ISO) 15189 accredited clinical laboratories, ultrasound, and x-ray facilities. There is no emergency department, and it does not receive referrals from other secondary institutions. The medical team comprised 2 consultants and 3 full-time medical officers, with support from clinical research fellows.

The EFSTH, located in Banjul, is the national teaching hospital receiving admissions from its Emergency Department and referrals from other hospitals serving the whole country. During the study, the pediatric unit had 73 beds and a medical team of 5 pediatric consultants and 10 junior medical staff. It has access to laboratory services offering full blood count analysis, blood grouping, and blood film microscopy for malaria parasites. Routine blood culture facilities were not consistently available onsite during the study, and blood cultures for study patients were processed at the MRCG at LSHTM site. The pediatric unit has an emergency department and offers 24-hour care supported by a radiology department with x-ray, ultrasound, and computer tomography scan facilities.

### Laboratory Methods

#### Blood Cultures

Blood samples, 1–3 mL, were collected into BD BACTEC PEDS PLUS/F culture vials for children aged up to 15 years, and into BD BACTEC Plus Aerobic/F* and Plus Anaerobic/F* culture bottles for those aged >15 years, by direct inoculation following manufacturer’s instructions. Children with suspected endocarditis had 3 sets of cultures taken. To ensure uniformity and prevention of contamination, collection followed a standard protocol where possible. Inoculated blood culture bottles were processed at the MRCG at LSHTM microbiology laboratory, optimally within 4 hours of collection; however, for a minority of blood samples collected out of hours at EFSTH, the samples were prestored in an incubator for up to 12 hours before transfer to the microbiology laboratories. Bacterial isolates were obtained using an automated blood-culture system BACTEC 9050 (Becton Dickinson, Temse, Belgium). In addition, blood culture vials were subcultured on agar plates before loading into the BACTEC machine, to minimize the risk of losing fastidious organisms.

Microbiological procedures were performed using standard media if bottles gave a positive signal within 5 days, otherwise they were reported negative. Further identification was done by cultural morphology, Gram’s staining, biochemical testing, and serological agglutination. *Staphylococcus* isolates were identified by coagulase, mannitol fermentation, and catalase tests. Nontyphoidal *Salmonella* and other Gram-negatives were identified using bioMérieux analytical profile index API 20 E (Becton Dickinson, Sparks, MD) and serotyped as previously described [28]. Antibiotic susceptibility was assessed using disc diffusion methods and interpreted according to Clinical and Laboratory Standards Institute (CLSI) interpretation guide for the years 2013 to 2015 [29]. For this study, all organisms found as normal skin or oral flora that were found only once were considered to be contaminants, including coagulase-negative staphylococci, alpha-hemolytic streptococci (other than *S pneumoniae*), and diphtheroids.

#### Urine Screen for Antibiotic Activity

Urine was collected from a subset of participants (those in whom clean-catch sampling was successful) before in-hospital antibiotic administration to assess frequency of prehospital antibiotic use. Antibiotic detection were performed according to standard methods [30]. Sterile filter paper discs coated with 20 μL of urine were placed on a Mueller-Hinton agar plate inoculated with antibiotic-sensitive *Staphylococcus aureus* (ATCC 25923 strain). After 18- to 24-hour incubation at 35–37°C, growth inhibition around the disc was considered evidence of antibiotic exposure. Commercial antibiotic susceptibility disks (penicillin, gentamicin, trimethoprim/sulfamethoxazole, and chloramphenicol) were used as positive controls, and sterile saline was used as negative control.

### Management of Patients

All patients were managed by pediatricians according to The Gambia National Standard Treatment Guideline and WHO Pocket Book of Hospital Care for Children [26]. Screening for malaria was done on admission with thick-film microscopy and treatment offered when confirmed. Standard first-line antimicrobials used in The Gambia for treatment of suspected sepsis in children are ampicillin or crystalline penicillin, plus gentamicin. For patients failing to respond to first-line antibiotic therapy, chloramphenicol is the usual antibiotic of choice and, where appropriate, ciprofloxacin. Ceftriaxone is usually reserved for severe sepsis.

### Statistical Analysis

Mean (standard deviation) was calculated for normally distributed variables and median (interquartile range [IQR]) for non-parametric variables. Cohen’s kappa coefficient was used to measure agreement between reported antibiotic use and urine antimicrobial activity. Fisher’s exact tests and χ ^2^ tests were used to compare categorical variables. Univariable binary logistic regression was used to determine the factors associated with bacteremia and mortality; intercepts were allowed for odds ratio (OR) calculations. When applying a multivariate logistic regression approach to bacteremia and mortality, a causal diagram was drawn before model building in both cases. To identify the most significant variables, initial models were built using all clinical variables, followed by a variable selection approach in which variables with the largest *P* values were iteratively excluded one at a time, until a handful of variables remained, and we have shown in Table 2 those with *P* < .05. Age and sex were retained because these were biologically known to affect the predictors as well as site of hospital of care. Prehospital antibiotic use was retained in the bacteremia model. Statistical calculations were performed in Genstat and R.

### Ethical Approval

Approval for the EUCLIDS study was obtained from the Joint MRCG at LSHTM-Gambia Government Ethics Committee (SCC 1287). A parent/guardian provided written (signed or thumb-printed) informed consent for their child to take part in the study. For parents not literate in English, an impartial witness was present throughout the informed consent discussion, undertaken in one of the local languages, and the impartial witness signed to attest to the completeness of the information given.

## RESULTS

### Patient Characteristics, Bacteremia Prevalence, and Urinary Screen for Antibiotic Activity

A total of 411 study participants were recruited, 57.6% (n = 237 of 411) at the EFSTH. Overall, 60.1% (n = 247 of 411) were male with a median age of 29 months (IQR, 11–82). A total of 74.9% (n = 308 of 411) reported up-to-date immunizations. Forty-two (13.3%) had a history of consanguinity, and 37 (9.0%) had a history of a comorbidity including sickle cell disease, cerebral palsy, asthma, congenital hydrocephalus, insulin dependent diabetes mellitus, epilepsy, and congenital and acquired heart diseases. A total of 12.2% (n = 50 of 411) had cigarette smoke exposure at home. A total of 3.4% (n = 14 of 411) had confirmed malaria infection. A total of 15.5% (n = 54 of 349) had severe wasting. A total of 30.2% (n = 124 of 411) were admitted at another healthcare facility before being seen at the study sites. The median duration of symptoms before presentation was 3.9 days (IQR, 2.64–7.18), and the median duration of stay at the study sites on admission was 6.5 days (IQR, 2.96–11.33).

Sixty-six bacterial pathogens were identified by blood culture from 65 of 411 (15.8%) study subjects, including 1 polymicrobial blood culture (*Escherichia coli* and *S pneumoniae*). Gram-positive organisms were more commonly identified than Gram-negative organisms (9.2% versus 6.8%; *P* ≤ .01). The most common isolated pathogen was *S aureus* (22, 5%), which was predominantly responsible for soft tissue and/or bone infections with or without sepsis. Table 1 summarizes the blood culture findings. Contaminants were present in 5.8% (n = 24 of 411). The clinical features of patients with and without bacteremia are shown in Supplementary Table S1.

**Table 1.** Blood Culture Isolates Stratified by Diagnostic Categorization^a^

		^b^Diagnostic Categorization						
Blood Culture Isolate		Stratified Prevalence (%)						
	Total (Column %)	Sepsis	Pneumonia	Meningitis	Soft Tissue	Bone	Other	Malaria Coinfection
Blood Culture Isolate	N = 411	N = 202	N = 144	N = 92	N = 43	N = 27	N = 30	N = 14
*Staphylococcus aureus*	22 (5.4)	17 (77.3)	2 (9.1)	2 (9.1)	8 (36.4)	4 (18.2)	2 (9.1)	0
*Streptococcus pneumoniae*	14 (3.4)	12 (85.7)	9 (64.3)	3 (21.4)	1 (7.1)	0 (0)	0 (0)	0
Other Gram positives	2 (0.5)	1 (50.0)	0 (0)	0 (0)	1 (50.0)	1 (50.0)	0 (0)	0
Sub-total: Gram-positive	38 (9.2)	30 (78.9)	11 (28.9)	5 (13.2)	10 (26.3)	5 (13.2)	2 (5.3)	0
Neisseria *meningitidis*	4 (1.0)	2 (50.0)	0 (0)	4 (100.0)	0 (0)	0 (0)	0 (0)	0
*Haemophilus influenzae* ^c^	6 (1.5)	5 (83.3)	2 (33.3)	3 (50.0)	0 (0)	1 (16.7)	0 (0)	0
*Escherichia coli*	4 (1.0)	3 (75.0)	2 (50.0)	0 (0)	0 (0)	0 (0)	0 (0)	0
*Pseudomonas* spp	3 (0.7)	2 (66.7)	1 (33.3)	0 (0)	0 (0)	0 (0)	0 (0)	0
*Salmonella* spp^d^	3 (0.7)	1 (33.3)	1 (33.3)	0 (0)	0 (0)	0 (0)	1 (33.3)	0
Other Gram negative	8 (1.9)	7 (87.5)	3 (37.5)	0 (0)	1 (12.5)	0 (0)	0 (0)	1
Sub-total: Gram-negatives	28 (6.8)	20 (71.4)	9 (32.1)	7 (25.0)	1 (3.6)	1 (3.6)	1 (3.6)	1
Total isolates	66 (16.1)	50 (75.8)	20 (30.3)	12 (18.2)	11 (16.7)	6 (9.1)	2 (3.0)	1

^a^The numbers of patients with blood culture-confirmed infection with common organisms, broken down into the common clinical syndrome groupings. Apart from the first (Total) column, percentages in brackets pertain to the proportion in that row, and may not add up to 100.0 because patients may have more than one syndrome. The “Other Gram-positives” row includes infection with *Streptococcus* Gp F and other *Streptococcus* spp. The Other Gram-negatives row includes infection with coliform species, including *Enterobacter cloacae*, *Klebsiella* spp, other *Enterobacter* spp, and unspecified Gram-negative organism.

^b^Diagnostic categorization variables are not mutually exclusive.

^c^All *Haemophilus influenzae* isolates were nontype B.

^d^All *Salmonella* species were identified as nontyphoidal.

Overall, 79.5% (n = 325 of 409) reported use of antibiotics in the 7 days before recruitment. Urine screens for antibiotic activity were completed on a subset of 113 recruits, 73 (64.6%) of whom reported prehospital antibiotic use in preceding 3 days; 80 (70.8%) had a positive screen for antibiotic activity (Supplementary Table S2). There was moderate agreement between reported history of antibiotic use and a positive screen for antibiotic activity (kappa 0.47). The 113 patients screened did not differ significantly in age from the overall cohort (32 vs 29 months; *P* = .32). A positive blood culture was found in 16 of 113 (14.2%) participants in whom the screen was performed. There was a trend towards increased bacteremia in patients with a positive urine antibiotic screen (OR = 2.9), but this was nonsignificant (*P* = .2; Fisher exact test).

### Antimicrobial Resistance

Antibiotic resistance was uncommon among the Gram-positive pathogens for commonly used first-line (7.9% [n = 3 of 38]; range, 7%–9%) and second-line (5.3% [n = 2 of 38]; range, 0%–14%) antimicrobials. Two cases of *Staphylococcus* and 1 case of *S pneumoniae* showed resistance to first-line antimicrobials. All Gram-positive organisms were sensitive to ceftriaxone. All * S aureus* isolates were methicillin-sensitive.

However, antibiotic resistance was common in Gram-negative pathogens for standard first-line (39.3% [n = 11 of 28]; range, 25%–100%) and second-line (46.4% [n = 13 of 28]; range, 33%–100%) antimicrobials. There was widespread resistance to trimethoprim/sulfamethoxazole in both Gram-positive (64.9% [n = 24 of 37]; range, 41%–100%) and Gram-negative (81.0% [n = 17 of 21]; range, 71%–100%) pathogens (see Supplementary Table S3).

### Factors Associated With Bacteremia and Mortality

A univariable analysis of clinical findings associated with bacteremia and mortality (Supplementary Table S1) found that of the significant (*P* < .05) variables, those with highest odds for bacteremia were meeting criteria for sepsis (OR, 5.4; 95% confidence interval [CI], 2.8–10.5) and bone infection (OR, 3.5; 95% CI, 1.5–8.0). The significant variables with highest odds for mortality were previous hospitalization (OR, 17.3; 95% CI, 8.5–35.2), altered consciousness (OR, 12.5; 95% CI, 6.5–24.2), and need for oxygen therapy (OR, 5.6; 95% CI, 3.0–10.4).

We used an iterative logistic regression approach to derive a minimal set of variables associated with bacteremia or mortality. Male gender, normal hydration status, and normal musculoskeletal exams on presentation were significantly less prevalent in the bacteremic patients. Sepsis, as defined by the international consensus definition [25], was a factor associated with bacteremia (see Table 2), whereas there was no association of bacteremia with malaria infection.

Mortality was 13.1% (n = 54 of 411) and 10.2% had sequelae at time of discharge (n = 42 of 411). The median age (IQR) of study participants who died or had sequelae at time of discharge was 16.3 (IQR, 5.9–60.0) and 56.0 (IQR, 16.6–111.1) months, respectively. Factors associated with mortality as analyzed in the multivariable model were a positive history of a comorbidity, clinical pallor, presence of tachypnoea, and altered consciousness. Tachycardia was protective against mortality in the multivariable logistic regression analysis (Table 2).

**Table 2.** Multivariable Analysis for Factors Associated With Bacteremia and Mortality^a^

Variable	Odds Ratio (95% CI)	*P* Value
Analysis for Bacteremia		
Male gender	0.37 (0.18–0.77)	.01
Admission to MRCG at LSHTM hospital	2.73 (1.28–5.82)	.01
Normal hydration status	0.31 (0.12–0.83)	.02
Normal musculoskeletal exam	0.13 (0.05–0.32)	<.01
Sepsis	12.75 (4.96–32.77)	<.01
Analysis for Mortality		
History of a comorbidity	3.66 (1.26–10.61)	.02
Clinical pallor	4.56 (1.45–14.40)	.01
Tachycardia for age	0.39 (0.18–0.84)	.02
Tachypnea for age	2.93 (1.22–7.01)	.02
Altered consciousness	7.15 (3.21–15.93)	<.01

Abbreviations: CI, confidence interval; MRCG at LSHTM, Medical Research Council The Gambia at London School of Hygiene & Tropical Medicine; OR, odds ratio.

^a^Clinical parameters were assessed on admission at the time of diagnosis. Top performing correlates of bacteremia and mortality were derived using a multivariate logistic regression approach, followed by a variable selection approach in which variables with the largest *P* values were iteratively excluded one at a time. The table includes those with those with *P* < .05.

## DISCUSSION

In this prospective, multicenter study of bacteremia amongst study subjects aged 1 month to 18 years presenting with sepsis or SFI to 2 hospitals in urban Gambia, we found a high prevalence of bacteremia. A total of 15.8% of all participants had an identifiable pathogen on blood culture, higher than that reported in similar studies in SSA [2, 6, 9, 10, 12, 21–23]. A factor that may have accounted for this high number was our strict inclusion criteria for sepsis and SFI. However, over 84% of participants with severe illness had no identifiable pathogen. The lack of viral and bacterial molecular diagnostics in this study as well as the very high rates of prehospital antibiotic use reported by parents, and confirmed by urinary screen for antibiotic activity, may therefore have led to an underestimate of the true frequency of bacterial etiology in this cohort.

We observed patterns of pathogen prevalence that were substantially different from previously published reports, highlighting the need for reassessing contemporary bacterial epidemiology to guide current and future practice. Combined mortality and severe morbidity associated with infection were substantial, affecting 23% of patients.

Multivariate analysis identified requirement for antimicrobial therapy on admission and requirement for immediate, aggressive management to prevent mortality as clinical features that predicted presence of bacteremia. Consistent with other recently published studies from SSA, *S aureus* was the predominant pathogen across all age groups [12, 14, 15, 31–34], except in the 2–5 age group where infections with *S pneumoniae* predominated. This contrasts with (1) a recent publication from Ghana in which nontyphoidal salmonella accounted for more than half of all invasive bacterial infections in children under 5 years of age [13] and (2) a study from The Gambia in 2007 in which *S pneumoniae* was the most common pathogen among all age groups [22].

One major factor that is likely to have contributed to this change in pathogen prevalence is the introduction of the pneumococcal conjugate vaccine (PCV) to the Gambian Expanded Program on Immunisation (EPI), with the 7-valent vaccine (PCV7) being introduced in 2009 followed by PCV13 in 2011. Recent surveillance studies, completed postintroduction of the Gambian PCV program, have shown a substantial reduction in invasive pneumococcal disease of approximately 55% among children aged 2 to 59 months [32]. Although the Gambian EPI is one of the most successful in SSA, with up to 10 vaccines being administered and with high vaccine coverage rates, it also has a poor record of completing vaccination schedules on time [35]. This factor may also have contributed to the high morbidity and mortality seen from vaccine-preventable infections in our cohort. Since the introduction of *H influenzae* type B (Hib) vaccine in The Gambia in 2007, rates of Hib disease have decreased, although there has been some recrudescence in Eastern Gambia [36]. Of the 6 cases of *H influenzae* sepsis identified in this study, none were serotype B.

Antibiotic resistance was found predominantly in Gram-negative organisms—a recognized and global concern[37]. These pathogens were often resistant to the first-line antimicrobials and at times resistant to ceftriaxone, which is usually reserved for meningitis, severe sepsis, or where other treatment options have failed; this may be a reflection on poor antibiotic stewardship in The Gambia. Gram-positive organisms were mostly susceptible to the first-line antimicrobials: crystalline penicillin/ampicillin and gentamicin. These continue to be the first-line antibiotics of choice as set out in the WHO Pocket Book of Hospital Care for Children [26], a commonly used resource in low-income settings for the clinical diagnosis and management of sick children. Given the increasing predominance of *S aureus* disease in SSA, and our findings that 73% of isolates were penicillin resistant, and 2 of 9 were gentamicin resistant, empiric regimens should treat bacteremia caused by *S aureus* (together with *S pneumoniae*, *H influenzae*, and *E coli*) effectively, for instance, using co-amoxiclav, because early adequate treatment of sepsis is key for improved survival. Although it was reassuring to see that all *S aureus* isolates were methicillin susceptible, close surveillance for emerging resistance remains crucial.

The management of severe sepsis should be guided by blood culture and antibiotic sensitivity testing, and this may lead to therapeutic options that include broader spectrum antibiotics such as piperacillin tazobactam. Maintaining informed choices for first- and second-line antibiotic prescribing will require ongoing microbiological surveillance and the development of antimicrobial stewardship strategies.

Trimethoprim/sulfamethoxazole is commonly used in The Gambia because of its broad-spectrum antimicrobial efficacy, low cost, and easy availability. However, we found that both Gram-negative and -positive bacteria were highly resistant to this antibiotic on laboratory testing, suggesting that trimethoprim/sulfamethoxazole in The Gambia, and probably elsewhere in SSA [38], should be reserved for prophylaxis and treatment of *Pneumocystis jirovecii* pneumonia.

Bacteremia was significantly more common in dehydrated children. This finding stresses the importance of assessment of hydration status and appropriate fluid resuscitation in suspected sepsis. Soft tissue, joint, and bone infections were associated with *S aureus*, indicating that all such cases need to be treated with antistaphylococcal antibiotics.

The risk of mortality was highest in those with comorbidities, including sickle cell disease or other congenital conditions, and in those with clinical features of pallor, tachypnoea, or altered consciousness. Therefore, the presence of these symptoms should be considered a “red flag”. They are recognizable by nursing and medical staff, and their importance is emphasized in the WHO Emergency Triage Assessment and Treatment (ETAT) guidelines [39]. Sickle cell disease was tested for in patients with anemia and detected in 9 patients. It was not a confounder for clinical pallor, because none of these patients were classified as having clinical pallor. Although dehydration was associated with bacteremia, tachycardia was associated with reduced mortality. Tachycardia may reflect hypovolemia caused by sepsis rather than dehydration, and it indicates maintained hemodynamic compensation. Further studies are needed to infer causality in these associations.

This study had strengths and limitations. We found high rates of reported prehospital antibiotic usage, confirmed by urinary screen for antibiotic activity in a subset of participants, which has implications for management of patients. This could have significantly affected the yield of positive isolates. Bacterial culture positivity rates may have been affected by storage of inoculated blood culture bottles at EFSTH before processing and the use of suboptimal blood volumes. Children up to age 15 years had blood collected in aerobic pediatric blood culture bottles, which are optimized for lower blood volumes. The storage and volume of blood are factors known to affect both culture yield—particularly for detection of rapidly growing bacteria including *S pneumoniae*—and the likelihood of contaminants [40]. Nonetheless, the rate of blood culture contamination (5.8% of blood cultures taken) is lower than in other SSA studies [12], and it suggests that poor technique is unlikely to have greatly influenced our results, reflecting the rigorous training undertaken by clinical staff. This study recruited only those children with sepsis or SFI, and thus the range of pathogens identified, and the rate of bacteremia, may not apply across the range of childhood infection. However, in an urban SSA environment where there is widespread and indiscriminate antibiotic use, this study has enabled us to identify bacterial pathogens and factors associated with morbidity and mortality. We found that antibiotic resistance to first-line antibiotics was more common in Gram-negative organisms; although the study focused on community-acquired infections, we cannot exclude the possibility that some tertiary referral patients had nosocomial infections, with a higher risk for resistance. Finally, the relatively small size of this study and number of bacterial pathogens identified has meant that we have been underpowered to evaluate the role of risk factors such as smoking and consanguinity on outcome.

## CONCLUSIONS

In conclusion, the bacteremia rate in Gambian pediatric patients with severe sepsis and SFI was high, despite high rates of prehospital antibiotic use. We observed a changing pattern of pathogen prevalence and antimicrobial resistance from previously published data, as well as subregional differences in prevalent pathogens. Our study highlights the need for structured bacterial surveillance programs in resource-limited settings, however scarce the resources may be.

## Supplementary Data

Supplementary materials are available at *Open Forum Infectious Diseases* online. Consisting of data provided by the authors to benefit the reader, the posted materials are not copyedited and are the sole responsibility of the authors, so questions or comments should be addressed to the corresponding author.

ofz332_suppl_supplementary_material
