# Supplementary material for: Bacteremia in Childhood Life-Threatening Infections in Urban Gambia: EUCLIDS in West Africa
Source: Open Forum Infect Dis. 2019 Jul 27;6(9):ofz332. doi: 10.1093/ofid/ofz332 (PMC6798247; doi:10.1093/ofid/ofz332)
Supplement: ofz332_suppl_supplementary_material [file ofz332_suppl_supplementary_material.pdf]

Supplementary Information

Bacteremia in childhood life-threatening infections in urban Gambia: EUCLIDS in West Africa

Secka F[1]*, Herberg JA[2]*, Sarr I[1], Darboe S[1], Sey G[1], Saidykhan M[1], Wathuo M[1], Kaforou, M[2], Antonio M[1], Roca A[1], Zaman SMA[1], Cebey-López M[3], Boeddha NP[4], Paulus S[5], Kohlfürst DS[6], Emonts M[7,8], Zenz W[6], Carrol ED[9], de Groot R[10], Schlapbach, L[11], Martinon-Torres F[3], Bojang K[1], Levin M[2+], van der Flier M[10+§], Anderson ST[1+] on behalf of the EUCLIDS consortium[%]

* denotes equal first author and [+]equal last author contributions

[%]The EUCLIDS consortium members are listed at the end of this section

Contents

Supplementary Table S1: Characteristics of bacteraemic and non-bacteremic patients, and univariable analysis for bacteremia and mortality 2

Supplementary Table S2 Presence of bacteremia in those with and without a positive urine screen for antibiotic activity 8

Supplementary Table S3 Antimicrobial resistance in isolates from bacteremic participants 9

Appendix: EUCLIDS CONSORTIUM MEMBERS 10

Supplementary Table S1: Characteristics of bacteraemic and non-bacteremic patients, and univariable analysis for bacteremia and mortality

Clinical Characteristic	Bacteremia (65 patients)	Non bacteremia (346 patients)	Univariable analysis for Bacteremia		Death (54 patients)	Home (357 patients)	Univariable analysis for Mortality	
	Number (%)	Number (%)	Odds ratio (95% CI)	p val	Number (%)	Number (%)	Odds ratio (95% CI)	p val
**Age group (411)**								
**0-11 months**	19 (29.2%)	89 (25.7%)	Reference group	0.89	23 (42.6%)	85 (23.8%)	Reference group	0.08
**11-23 months**	14 (21.5%)	58 (16.8%)	1.13 (0.53-2.43)		8 (14.8%)	64 (17.9%)	0.46 (0.19-1.10)	
**24-59 months**	13 (20.0%)	75 (21.7%)	0.81 (0.38-1.75)		7 (16.7%)	79 (22.1%)	0.42 (0.18-0.96)	
**60-119 months**	12 (18.5%)	78 (22.5%)	0.72 (0.33-1.58)		8 (14.8%)	82 (23.0%)	0.36 (0.15-0.85)	
**120-143 months**	3 (4.6%)	19 (5.5%)	0.74 (0.20-2.75)		1 (1.9%)	21 (5.9%)	0.18 (0.02-1.36)	
**144- months**	4 (6.2%)	27 (7.8%)	0.69 (0.22-2.22)		5 (9.3%)	26 (7.3%)	0.71 (0.25-2.05)	
**Male Gender (409)**	34 (52.3%)	212 (61.3%)	0.68 (0.40-1.16)	0.16	33(61.1%)	213 (59.7%)	1.05 (0.58-1.88)	0.88
**Ethnicity (411)**								
**Fula**	14 (21.5%)	57 (16.5%)	Reference group	0.59	10 (18.5%)	61 (17.1%)	Reference group	0.19
**Jola**	10 (15.4%)	52 (15.0%)	0.78 (0.32-1.91)		12 (22.2%)	50 (14.0%)	1.46 (0.58-3.67)	
**Mandinka**	30 (46.2%)	156 (45.1%)	0.78 (0.39-1.58)		17 (31.5%)	169 (47.3%)	0.61 (0.27-1.41)	
**Wolof**	2 (3.1%)	30 (8.7%)	0.27 (0.06-1.27)		3 (5.6%)	29 (8.1%)	0.63 (0.16-2.4)	
**Other tribe**	9 (13.8%)	51 (14.7%)	0.72 (0.29-1.80)		12 (22.2%)	48 (13.4%)	1.53 (0.61-3.83)	

Clinical Characteristic	Bacteremia (65 patients)	Non bacteremia (346 patients)	Univariable analysis for Bacteremia		Death (54 patients)	Home (357 patients)	Univariable analysis for Mortality	
	Number (%)	Number (%)	Odds ratio (95% CI)	p val	Number (%)	Number (%)	Odds ratio (95% CI)	p val
**Dry Season (Nov –May) (411)**	37 (56.9%)	188 (54.3%)	1.11 (0.65-1.90)	0.70	26 (48.1%)	199 (55.7)	0.74 (0.42-1.31)	0.30
**Previous severe infection (394)**	5 (7.7%)	32 (9.2%)	0.79 (0.30- 2.11)	0.64	5 (9.3%)	32 (9.0%)	1.01 (0.37-2.71)	0.84
**Prematurity (396)**	0 (0%)	3 (0.9%)	0.01 (0.01-0.01)	0.60	1 (1.9%)	2 (0.6%)	3.35 (0.30-37.44)	0.59
**Consanguinity (251)**	10 (12.4%)	43 (15.4%)	1.206 (0.55-2.64)	0.64	5 (9.3%)	48 (13.4%)	0.55 (0.30-1.01)	0.14
**History of a co-morbidity (411)**	3 (4.6%)	35 (10.1%)	0.44 (0.13-1.49)	0.19	8 (14.8%)	29 (8.1%)	1.83 (0.88-3.80)	0.11
**Immunization up to date (328)**	45 (69.2%)	263 (76.0%)	0.68 (0.22-2.14)	0.51	45 (83.3%)	236 (73.7%)	3.25 (0.43-24.71)	0.32
**Cigarette exposure (250)**	9 (13.8%)	54 (15.6%)	0.78 (0.35-1.73)	0.54	6 (11.1%)	57 (16.0%)	0.60 (0.24-1.52)	0.53
**Other hospitalization before study admission (411)**	19 (29.2%)	105 (30.3%)	0.95 (0.53-1.70)	0.86	5 (7.7%)	32 (9.2%)	17.28 (8.46-35.29)	<0.01
**LOS at local hospital (123 of 124)**								
**2 days and less**	14 (73.7%)	71 (68.3%)	1.04 (0.54-1.99)	0.89	16 (29.6%)	61 (17.1%)	17.91 (7.60-42.21)	<0.01
**>2 days**	5 (26.3%)	33 (31.7%)	0.80 (0.30-2.15)		13 (24.1%)	33 (9.2%)	24.47 (5.94-100.8)	
**Symptom days at presentation (293)**								
**2 days and less**	3 (6.5%)	38 (15.4%)	0.41 (0.12-1.47)	0.39	3 (5.6%)	25 (7.0%)	1.17 (0.35-3.95)	0.24
**3-7 days**	27 (58.7%)	144 (58.3%)	0.98 (0.52-1.85)		(%)	(%)	2.03 (0.94-4.36)	
**>7days**	16 (34.8%)	65 (26.3%)	1.28 (0.62-2.67)		13 (24.1%)	72 (20.2%)	2.07 (0.86-4.97)	

Clinical Characteristic	Bacteremia (65 patients)	Non bacteremia (346 patients)	Univariable analysis for Bacteremia		Death (54 patients)	Home (357 patients)	Univariable analysis for Mortality	
	Number (%)	Number (%)	Odds ratio (95% CI)	p val	Number (%)	Number (%)	Odds ratio (95% CI)	p val
**Pre-hospital antibiotics within 3 days (409)**	54 (83.1%)	271 (78.3%)	1.32 (0.66-2.66)	0.43	46(85.2%)	279(78.2%)	1.57 (0.71-3.46)	0.27
**Admitted MRCG at LSHTM hospital (411)**	34 (52.3%)	140 (40.5%)	1.61 (0.95-2.75)	0.08	29(53.7%)	95(26.6%)	0.17 (0.08-0.38)	<0.01
**Severe Wasting WLZ score <-3SD (349)**	11 (16.9%)	43 (12.4%)	1.35 (0.65-2.80)	0.42	7 (13.0%)	47 (13.2%)	0.90 (0.38-2.12)	0.80
**Severe Underweight WAZ score <-3SD (358)**	10 (15.4%)	42 (12.1%)	1.25 (0.59-2.65)	0.56	6 (11.1%)	46 (12.9%)	0.80 (0.32-1.98)	0.63
**Severe Stunting LAZ score <-3SD (350)**	9 (13.8%)	23 (6.6%)	2.15 (0.94-4.91)	0.07	4 (7.4%)	28 (7.8%)	0.87 (0.29-2.59)	0.80
**Temperature (411)**								
**35.0-38.4˚C**	42 (64.6%)	208 (60.1%)	Reference group	0.76	30 (55.6%)	220 (61.6%)	Reference group	0.27
**38.5 -39.5˚C**	18 (27.7%)	112 (32.4%)	0.80 (0.44-1.45)		17 (31.5%)	113 (31.7%)	1.10 (0.58-2.09)	
**>39.5˚C**	5 (7.7%)	26 (7.5%)	0.95 (0.35-2.62)		7 (13.0%)	24 (6.7%)	2.14 (0.85-5.38)	
**Clinical Pallor (411)**	5 (7.7%)	25 (7.2%)	1.07 (0.39-2.90)	0.89	8 (14.8%)	22 (6.2%)	2.65 (1.12-6.28)	0.03
**Normal hydration status (405)**	53 (81.5%)	313 (90.5%)	0.49 (0.23-1.06)	0.07	46 (85.2%)	320 (89.6%)	0.56 (0.24-1.28)	0.17

Clinical Characteristic	Bacteremia (65 patients)	Non bacteremia (346 patients)	Univariable analysis for Bacteremia		Death (54 patients)	Home (357 patients)	Univariable analysis for Mortality	
	Number (%)	Number (%)	Odds ratio (95% CI)	p val	Number (%)	Number (%)	Odds ratio (95% CI)	p val
**Central cyanosis (400)**	2 (3.1%)	21 (6.1%)	0.48 (0.11-2.12)	0.34	2 (3.1%)	21 (6.1%)	1.19 (0.50-2.81)	0.78
**Tachycardia for age (411)**	31 (47.7%)	183 (52.9%)	0.81 (0.48-1.38)	0.44	22 (40.7%)	192 (53.8%)	0.59 (0.33-1.06)	0.08
**Tachypnea for age (408)**	38 (58.5%)	220 (63.6%)	0.82 (0.48-1.42)	0.49	42 (77.8%)	216 (60.5%)	2.24 (1.14-4.40)	0.02
**Active Skin Infection (409)**	9 (13.8%)	31 (9.0%)	1.62 (0.73-3.59)	0.23	6 (11.1%)	34 (9.5%)	1.10 (0.57-2.13)	0.78
**No Palpable Lymphadenopathy (399)**	57 (87.7%)	295 (85.3%)	1.32 (0.54-3.26)	0.55	46 (85.2%)	295 (82.6%)	1.00 (0.41-2.46)	0.99
**No Hepatomegaly and/or Spleno-megaly (401)**	52 (80.0%)	296 (85.5%)	0.67 (0.34-1.32)	0.24	41 (75.9%)	297 (83.2%)	0.59 (0.298-1.19)	0.14
**Altered Consciousness (403)**	14 (21.5%)	85 (24.6%)	1.19 (0.63-2.27)	0.59	39 (72.2%)	60 (16.8%)	12.52 (6.49-24.16)	<0.01
**Seizures prior to/or on admission (395)**	9 (13.8%)	85 (24.6%)	0.50 (0.23-1.05)	0.07	26 (48.1%)	68 (19.0%)	4.04 (2.21-7.40)	<0.01
**Abnormal focal neurology (392)**	2 (3.1%)	18 (5.2%)	0.57 (0.13-2.50)	0.45	8 (14.8%)	12 (3.4%)	4.73 (1.84-12.17)	<0.01
**Normal cardiovascular examination (399)**	58 (89.2%)	330 (95.4%)	0.47 (0.12-1.81)	0.27	48 (88.9%)	340 (95.2%)	1.18 (0.35-3.91)	0.79
**Normal musculoskeletal exams (403)**	21 (32.3%)	50 (14.5%)	0.34 (0.18-0.61)	<0.01	45 (83.3%)	287 (80.4%)	1.43 (0.62-3.32)	0.40
**No malaria parasitemia (387)**	64 (98.5%)	333 (96.2%)	2.64 (0.34-20.56)	0.35	51 (94.4%)	322 (90.2%)	0.95 (0.21-4.37)	0.97

Clinical Characteristic	Bacteremia (65 patients)	Non bacteremia (346 patients)	Univariable analysis for Bacteremia		Death (54 patients)	Home (357 patients)	Univariable analysis for Mortality	
	Number (%)	Number (%)	Odds ratio (95% CI)	p val	Number (%)	Number (%)	Odds ratio (95% CI)	p val
**Hemoglobin <8g/dl (398)**	13 (20.0%)	65 (18.8%)	1.08 (0.55-2.10)	0.82	10 (18.5%)	68 (19.0 %)	0.97 (0.47-2.03)	0.94
**White Blood Cells X 10^9/L (402)**							-	-
**1.7-15.0**	21 (32.8%)	119 (35.2%)	1.41 (0.17-11.88)	0.93	24 (44.4%)	116 (32.5%)		
**>15.0-25.0**	21 (32.8%)	121 (35.8%)	1.39 (0.17 (11.68)		16 (29.6%)	126 (35.3%)		
**>25.0-35.0**	13 (20.3%)	58 (17.2%)	1.79 (0.21-15.61)		12 (22.2%)	59 (16.5%)		
**>35.0**	9 (14.1%)	40 (11.8%)	1.8 (0.20-16.26)		2 (3.7%)	47 (13.2%)		
**Neutrophil count X 10^9/L (400)**							-	-
**0.1-10.0**	18 (28.6%)	131 (38.9%)	0.62 (0.12-3.09)	0.30	27 (50.0%)	122 (34.2%)		
**>10.0-20.0**	23 (36.5%)	121 (35.9%)	0.86 (0.17-4.21)		17 (31.5%)	127 (35.6%)		
**>20.0-30.0**	18 (28.6%)	58 (17.2%)	1.3990.27-7.05)		8 (14.8%)	64 (17.9%)		
**>30.0**	4 (6.3%)	27 (8.0%)	0.75 (0.12-4.53)		2 (3.7%)	33 (9.2%)		
**Lymphocytes x 10^9/L (400)**							-	-
**0.6-5.0**	43 (67.2%)	227 (67.6%)	1.89 (0.24-15.18)	0.88	37 (68.5%)	233 (65.3%)		
**>5.0-10.0**	18 (28.1%)	88 (26.2%)	2.05 (0.25-16.99)		12 (22.2%)	94 (26.3%)		
**>10.0**	3 (4.7%)	21 (6.2%)	1.43 (0.13-15.5)		5 (9.3%)	19 (5.3%)		

Clinical Characteristic	Bacteremia (65 patients)	Non bacteremia (346 patients)	Univariable analysis for Bacteremia		Death (54 patients)	Home (357 patients)	Univariable analysis for Mortality	
	Number (%)	Number (%)	Odds ratio (95% CI)	p val	Number (%)	Number (%)	Odds ratio (95% CI)	p val
**Platelets x 10^9/L (402)**							-	-
**21-<140**	13 (20.3%)	39 (11.6%)	2.69 (0.30-22.78)	0.32	12(22.2%)	41 (11.5%)		
**140-400**	27 (42.2%)	160 (47.5%)	1.35 (0.16-11.23)		19 (35.2%)	168 (47.1%)		
**>400**	24 (37.5%)	138 (40.9%)	1.39 (0.17-11.63)		23 (42.6%)	139 (38.9%)		
**Need for Oxygen therapy (386)**	17 (26.2%)	89 (25.7%)	1.03 (0.56-1.89)	0.94	32 (59.3%)	73 (20.4%)	5.62 (3.04-10.40)	<0.01
**Meeting criteria for sepsis (401)**	52 (80.0%)	150 (43.4%)	5.4 (2.79-10.48)	<0.01	39 (72.2%)	163 (45.7%)	2.94 (1.56-5.52)	<0.01
**Bone Infection (411)**	10 (15.4%)	17 (4.9%)	3.52 (1.53-8.08)	<0.01	2 (3.7%)	25 (7.0%)	0.66 (0.30-1.45)	0.30
**Soft tissue infection (411)**	10 (15.4%)	33 (9.5%)	0.58 (0.27-1.24)	0.16	3 (5.6%)	40 (11.2%)	0.47 (0.14-1.56)	0.21
**Meningitis (411)**	12 (18.5%)	80 (23.1%)	1.33 (0.68-2.61)	<0.01	17 (31.5%)	75 (21.0%)	1.72 (0.93-3.22)	0.09
**Pneumonia (411)**	20 (30.8%)	124 (35.8%)	0.80 (0.45 – 1.41)	0.43	10 (18.5%)	134 (37.5%)	0.38 (0.18-0.78)	<0.01
**Other infections (411)**	7 (10.8%)	23 (6.6%)	1.70 (0.70-4.12)	0.25	5 (9.3%)	25 (7.0%)	1.36 (0.50-3.70)	0.55

Abbreviations: WLZ – weight for length Z score, WAZ – weight for age Z score, LAZ – length for age Z score, LOS – length of stay.

The table shows demographic and clinical features of patients in the bacteremia vs non-bacteremia groups, and results for univariable analysis of clinical variables for the outcomes of bacteremia, and mortality. Clinical assessments reflected those at time of initial presentation to the study site and for most participants this coincided with enrolment. Missing values were observed for some variables, and the number of patients for whom data were analyzed is shown in parenthesis in the Clinical Characteristic column.

Supplementary Table S2 Presence of bacteremia in those with and without a positive urine screen for antibiotic activity

	Bacteremia	No bacteremia	Total
Positive urine screen	14*	76	80
Negative urine screen	2§	31	33
Total	16	97	**113**

* 8 Gram-positive (4 *S.aureus*, 2 *Strep. pneumoniae*, 1 Streptococcus Group G, 1 Streptococcus spp) and 6 Gram-negative (coliform, *E.coli*, *H.influenzae* (non type B), *N.meningitidis*, *Pseudomonas* spp, *Salmonella* spp).

§ All gram positive: 2 *S.aureus*

The 80 patients with a positive urine screen for antibiotic activity included 33 of 48 participants from the EFSTH and 47 of 65 from the MRCG at LSHTM hospital. There was no significant difference in proportion of bacteremic patients between groups with a positive and negative urine screen for antibiotic activity (*p=0.2*, Fisher Exact test). Odds Ratio for bacteremia if you had a positive vs negative urine antibiotic screen was 2.86.

Supplementary Table S3 Antimicrobial resistance in isolates from bacteremic participants

Bacterial pathogen in blood culture	No. of isolates	Antibiotic resistance to different antibiotics tested: number resistant (% of isolates)											
		Amp	Eryth	Gent	Cotrim	Pen	Ceftriax	Chlor	Cefurox	Cipro	Cloxa	Ceftaz	Vanc
S. aureus	22	-	2(9)	2(9)	9(41)	16(73)	0	0	0	0	0	-	0
S. pneumoniae	14	0	0	1(100)	14(100)	1(7)	0	2(14)	-	0	-	-	-
Strep Group F	1	0	0	-	-	0	0	0	-	-	-	-	0
Other *Streptococcus* Spp	1	0	-	0	1(100)	0	0	0	0	0	-	-	-
**Total Gram Positives**	**38**												
N. meningitidis	4	0	4(100)	-	4(100)	0	0	0	0	-	-	0	-
H. influenzae	6	0	-	-	4(80)	-	0	2(33)	0	0	-	1(20)	-
Coliform species	2	2(100)	-	0	2(100)	-	0	0	1(50)	0	-	0	-
E. coli	4	3(75)	-	1(25)	4(100)	-	0	2(50)	0	0	-	0	-
Enterobacter cloacae	1	1(100)	-	1(100)	-	-	-	1(100)	0	0	-	-	-
Other *Enterococcus* Spp.	1	0	-	-	0	1(100)	-	0	-	1(100)	-	-	0
Unspecified Gram neg	1	1(100)	-	-	1(100)	1(100)	-	0	1(100)	1(100)	-	1(100)	-
K. pneumoniae	3	3(100)	-	2(67)	2(67)	-	1(50)	2(67)	2(67)	1(33)	-	1(50)	-
Pseudo. aeruginosa	1	-	-	0	-	-	0	-	1(100)	-	-	-	-
Pseudo. fluorescence	1	-	-	0	-	-	-	-	1(100)	0	-	-	-
Pseudo. inteola	1	1(100)	-	0	-	-	-	-	-	0	-	0	-
Salmonella spp.	3	0	-	-	0	1(33)	0	0	0	0	-	-	-
**Total Gram Negatives**	**28**												

Amp=Ampicillin, Eryth=Erythromycin, Gent= Gentamycin, Cotrim=Clotrimoxazole, Pen= Penicillin, Ceftriax=Ceftriazone, Chlor=Cloramphenicol, Cefurox=Cefuroxime, Cipro=Ciprofloxacin, Cloxa=Cloxacillin, Ceftaz=Ceftazidine, Vanc=Vancomycin

Table shows breakdown of antibiotic resistance results in bacterial isolates from blood culture-positive patients

Appendix: EUCLIDS CONSORTIUM MEMBERS

EUCLIDS consortium (www.euclids-project.eu) is composed by:

<u>Imperial College partner (UK)</u>

Members of the EUCLIDS Consortium at Imperial College London (UK)

Principal and co-investigators

<u>Michael Levin (grant application, EUCLIDS Coordinator)</u>
Dr. Lachlan Coin (bioinformatics)
Stuart Gormley (clinical coordination)
Shea Hamilton (proteomics)
Jethro Herberg (grant application, PI)
Bernardo Hourmat (project management)
Clive Hoggart (statistical genomics)
Myrsini Kaforou (bioinformatics)
Vanessa Sancho-Shimizu (genetics)
Victoria Wright (grant application, scientific coordination)

<u>Consortium members at Imperial College</u>
Amina Abdulla
Paul Agapow
Maeve Bartlett
Evangelos Bellos
Hariklia Eleftherohorinou
Rachel Galassini
David Inwald
Meg Mashbat
Stefanie Menikou
Sobia Mustafa
Simon Nadel
Rahmeen Rahman
Clare Thakker

EUCLIDS UK Clinical Network

Poole Hospital NHS Foundation Trust, Poole: Dr S Bokhandi (PI), Sue Power, Heather Barham

Cambridge University Hospitals NHS Trust, Cambridge: Dr N Pathan (PI), Jenna Ridout, Deborah White, Sarah Thurston

University Hospital Southampton, Southampton: Prof S Faust (PI), Dr S Patel (co-investigator), Jenni McCorkell.

Nottingham University Hospital NHS Trust: Dr P Davies (PI), Lindsey Crate, Helen Navarra, Stephanie Carter

University Hospitals of Leicester NHS Trust, Leicester: Dr R Ramaiah (PI), Rekha Patel

Portsmouth Hospitals NHS Trust, London: Dr Catherine Tuffrey (PI), Andrew Gribbin, Sharon McCready

Great Ormond Street Hospital, London: Dr Mark Peters (PI), Katie Hardy, Fran Standing, Lauren O’Neill, Eugenia Abelake

King’s College Hospital NHS Foundation Trust, London; Dr Akash Deep (PI), Eniola Nsirim

Oxford University Hospitals NHS Foundation Trust, Oxford Prof A Pollard (PI), Louise Willis, Zoe Young

Kettering General Hospital NHS Foundation Trust, Kettering: Dr C Royad (PI), Sonia White

Central Manchester NHS Trust, Manchester: Dr PM Fortune (PI), Phil Hudnott

SERGAS Partner (Spain)

Principal Investigators
Federico Martinón-Torres[1]
Antonio Salas[1,2]

GENVIP RESEARCH GROUP (in alphabetical order):
Fernando Álvez González[1], Ruth Barral-Arca[1,2], Miriam Cebey-López[1], María José Curras-Tuala[1,2], Natalia García[1], Luisa García Vicente[1], Alberto Gómez-Carballa[1,2], Jose Gómez Rial[1], Andrea Grela Beiroa[1], Antonio Justicia Grande[1], Pilar Leboráns Iglesias[1], Alba Elena Martínez Santos[1], Federico Martinón-Torres[1], Nazareth Martinón-Torres[1], José María Martinón Sánchez[1], Beatriz Morillo Gutiérrez[1], Belén Mosquera Pérez[1], Pablo Obando Pacheco[1], Jacobo Pardo-Seco[1,2], Sara Pischedda[1,2], Irene Rivero Calle[1], Carmen Rodríguez-Tenreiro[1], Lorenzo Redondo-Collazo[1], Antonio Salas Ellacuriaga[1,2], Sonia Serén Fernández[1], María del Sol Porto Silva[1], Ana Vega[1,3,] Lucía Vilanova Trillo[1].
[1] Translational Pediatrics and Infectious Diseases, Pediatrics Department, Hospital Clínico Universitario de Santiago, Santiago de Compostela, Spain, and GENVIP Research Group (www.genvip.org), Instituto de Investigación Sanitaria de Santiago, Galicia, Spain.
[2] Unidade de Xenética, Departamento de Anatomía Patolóxica e Ciencias Forenses, Instituto de Ciencias Forenses, Facultade de Medicina, Universidade de Santiago de Compostela, and GenPop Research Group, Instituto de Investigaciones Sanitarias (IDIS), Hospital Clínico Universitario de Santiago, Galicia, Spain
[3] Fundación Pública Galega de Medicina Xenómica, Servizo Galego de Saúde (SERGAS), Instituto de Investigaciones Sanitarias (IDIS), and Grupo de Medicina Xenómica, Centro de Investigación Biomédica en Red de Enfermedades Raras (CIBERER), Universidade de Santiago de Compostela (USC), Santiago de Compostela, Spain

EUCLIDS SPANISH CLINICAL NETWORK:
Susana Beatriz Reyes[1], María Cruz León León[1], Álvaro Navarro Mingorance[1], Xavier Gabaldó Barrios[1], Eider Oñate Vergara[2], Andrés Concha Torre[3], Ana Vivanco[3], Reyes Fernández[3], Francisco Giménez Sánchez[4], Miguel Sánchez Forte[4], Pablo Rojo[5], J.Ruiz Contreras[5], Alba Palacios[5], Cristina Epalza Ibarrondo[5], Elizabeth Fernández Cooke[5], Marisa Navarro[6], Cristina Álvarez Álvarez[6], María José Lozano[6], Eduardo Carreras[7], Sonia Brió Sanagustín[7], Olaf Neth[8], Mª del Carmen Martínez Padilla[9], Luis Manuel Prieto Tato[10], Sara Guillén[10], Laura Fernández Silveira[11], David Moreno[12].
[1] Hospital Clínico Universitario Virgen de la Arrixaca; Murcia, Spain.
[2] Hospital de Donostia; San Sebastián, Spain.
[3] Hospital Universitario Central de Asturias; Asturias, Spain.
[4] Complejo Hospitalario Torrecárdenas; Almería, Spain.
[5] Hospital Universitario 12 de Octubre; Madrid, Spain.
[6] Hospital General Universitario Gregorio Marañón; Madrid, Spain.

[7] Hospital de la Santa Creu i Sant Pau; Barcelona, Spain.
[8] Hospital Universitario Virgen del Rocío; Sevilla, Spain.
[9] Complejo Hospitalario de Jaén; Jaén, Spain.
[10] Hospital Universitario de Getafe; Madrid, Spain.
[11] Hospital Universitario y Politécnico de La Fe; Valencia, Spain.
[12] Hospital Regional Universitario Carlos Haya; Málaga, Spain.

Members of the Pediatric Dutch Bacterial Infection Genetics (PeD-BIG) network (the Netherlands)

Steering committee:
Coordination: R. de Groot [1], A.M. Tutu van Furth [2], M. van der Flier [1]
Coordination Intensive Care: N.P. Boeddha [3], G.J.A. Driessen [3], M. Emonts [3, 4, 5], J.A. Hazelzet [3]
Other members: T.W. Kuijpers [7], D. Pajkrt [7], E.A.M. Sanders [6] , D. van de Beek [8], A. van der Ende [8]
Trial coordinator: H.L.A. Philipsen [1]

Local investigators (in alphabetical order)
A.O.A. Adeel [9], M.A. Breukels [10], D.M.C. Brinkman [11], C.C.M.M. de Korte [12], E. de Vries [13], W.J. de Waal [15], R. Dekkers [15], A. Dings-Lammertink [16], R.A. Doedens [17], A.E. Donker [18], M. Dousma[19], T.E. Faber [20], G.P.J.M. Gerrits[21], J.A.M. Gerver [22], J. Heidema [23], J. Homan-van der Veen [24], M.A.M. Jacobs [25], N.J.G. Jansen [6], P. Kawczynski [26], K. Klucovska [27], M.C.J. Kneyber [28], Y. Koopman-Keemink [29], V.J. Langenhorst [30], J. Leusink [31], B.F. Loza [32], I.T. Merth [33], C.J. Miedema [34], C. Neeleman [1], J.G. Noordzij [35], C.C. Obihara [36], A.L.T. van Overbeek – van Gils [37], G.H. Poortman [38],S.T. Potgieter [39], J. Potjewijd [40], P.P.R. Rosias [41], T. Sprong [21], G.W. ten Tussher [42], B.J. Thio [43], G.A. Tramper-Stranders [44], M. van Deuren [1], H. van der Meer [2], A.J.M. van Kuppevelt [45], A.M. van Wermeskerken [46], W.A. Verwijs [47], T.F.W. Wolfs [4].

1. Radboud University Medical Center – Amalia Children's Hospital, Nijmegen, The Netherlands
2. Vrije Universiteit University Medical Center, Amsterdam, The Netherlands
3. Erasmus Medical Center – Sophia Children's Hospital, Rotterdam, The Netherlands
4. Institute of Cellular Medicine, Newcastle University, Newcastle upon Tyne, United Kingdom
5. Paediatric Infectious Diseases and Immunology Department, Newcastle upon Tyne Hospitals Foundation Trust, Great North Children's Hospital, Newcastle upon Tyne, United Kingdom
6. University Medical Center Utrecht – Wilhelmina Children's Hospital, Utrecht, The Netherlands
7. Academic Medical Center – Emma Children's Hospital, University of Amsterdam, Amsterdam, The Netherlands
8. Academic Medical Center, University of Amsterdam, Amsterdam, The Netherlands
9. Kennemer Gasthuis, Haarlem, The Netherlands
10. Elkerliek Hospital, Helmond, The Netherlands
11. Alrijne Hospital, Leiderdorp, The Netherlands
12. Beatrix Hospital, Gorinchem, The Netherlands
13. Jeroen Bosch Hospital, 's-Hertogenbosch, The Netherlands
14. Diakonessenhuis, Utrecht, The Netherlands
15. Maasziekenhuis Pantein, Boxmeer, The Netherlands
16. Gelre Hospitals, Zutphen, The Netherlands
17. Martini Hospital, Groningen, The Netherlands

[18.] Maxima Medical Center, Veldhoven, The Netherlands
[19.] Gemini Hospital, Den Helder, The Netherlands
[20.] Medical Center Leeuwarden, Leeuwarden, The Netherlands
[21.] Canisius-Wilhelmina Hospital, Nijmegen, The Netherlands
[22.] Rode Kruis Hospital, Beverwijk, The Netherlands
[23.] St. Antonius Hospital, Nieuwegein, The Netherlands
[24.] Deventer Hospital, Deventer, The Netherlands
[25.] Slingeland Hospital, Doetinchem, The Netherlands
[26.] Refaja Hospital, Stadskanaal, The Netherlands
[27.] Bethesda Hospital, Hoogeveen, The Netherlands
[28.] University Medical Center Groningen, Beatrix Children's hospital, Groningen, The Netherlands
[29.] Haga Hospital – Juliana Children's Hospital, Den Haag, The Netherlands
[30.] Isala Hospital, Zwolle, The Netherlands
[31.] Bernhoven Hospital, Uden, The Netherlands
[32.] VieCuri Medical Center, Venlo, The Netherlands
[33.] Ziekenhuisgroep Twente, Almelo-Hengelo, The Netherlands
[34.] Catharina Hospital, Eindhoven, The Netherlands
[35.] Reinier de Graaf Gasthuis, Delft, The Netherlands
[36.] ETZ Elisabeth, Tilburg, The Netherlands
[37.] Scheper Hospital, Emmen, The Netherlands
[38.] St. Jansdal Hospital, Hardewijk, The Netherlands
[39.] Laurentius Hospital, Roermond, The Netherlands
[40.] Isala Diaconessenhuis, Meppel, The Netherlands
[41.] Zuyderland Medical Center, Sittard-Geleen, The Netherlands
[42.] Westfriesgasthuis, Hoorn, The Netherlands
[43.] Medisch Spectrum Twente, Enschede, The Netherlands
[44.] St. Franciscus Gasthuis, Rotterdam, The Netherlands
[45.] Streekziekenhuis Koningin Beatrix, Winterswijk, The Netherlands
[46.] Flevo Hospital, Almere, The Netherlands
[47.] Zuwe Hofpoort Hospital, Woerden, The Netherlands

Swiss Pediatric Sepsis Study

Steering Committee*: Luregn J Schlapbach, MD, FCICM [1,2,3], Philipp Agyeman, MD [1], Christoph Aebi, MD [1], Christoph Berger, MD [1]*

Luregn J Schlapbach, MD, FCICM [1,2,3], Philipp Agyeman, MD [1], Christoph Aebi, MD [1], Eric Giannoni, MD [4,5], Martin Stocker, MD [6], Klara M Posfay-Barbe, MD [7], Ulrich Heininger, MD [8], Sara Bernhard-Stirnemann, MD [9], Anita Niederer-Loher, MD [10], Christian Kahlert, MD [10], Paul Hasters, MD [11], Christa Relly, MD [12], Walter Baer, MD [13], Christoph Berger, MD [12] **for the Swiss Pediatric Sepsis Study**

[1.] Department of Pediatrics, Inselspital, Bern University Hospital, University of Bern, Switzerland
[2.] Paediatric Critical Care Research Group, Mater Research Institute, University of Queensland, Brisbane, Australia
[3.] Paediatric Intensive Care Unit, Lady Cilento Children's Hospital, Children's Health Queensland, Brisbane, Australia
[4.] Service of Neonatology, Lausanne University Hospital, Lausanne, Switzerland

5. Infectious Diseases Service, Lausanne University Hospital, Lausanne, Switzerland
6. Department of Pediatrics, Children's Hospital Lucerne, Lucerne, Switzerland
7. Pediatric Infectious Diseases Unit, Children's Hospital of Geneva, University Hospitals of Geneva, Geneva, Switzerland
8. Infectious Diseases and Vaccinology, University of Basel Children's Hospital, Basel, Switzerland
9. Children's Hospital Aarau, Aarau, Switzerland
10. Division of Infectious Diseases and Hospital Epidemiology, Children's Hospital of Eastern Switzerland St. Gallen, St. Gallen, Switzerland
11. Department of Neonatology, University Hospital Zurich, Zurich, Switzerland
12. Division of Infectious Diseases and Hospital Epidemiology, and Children's Research Center, University Children's Hospital Zurich, Switzerland
13. Children's Hospital Chur, Chur, Switzerland

Liverpool Partner

Principal Investigators
Enitan Carrol[1]
Stéphane Paulus [1,2]

ALDER HEY SERIOUS PAEDIATRIC INFECTION RESEARCH GROUP (ASPIRE) (in alphabetical order):
Hannah Frederick[3], Rebecca Jennings[3], Joanne Johnston[3], Rhian Kenwright[3]

[1] Department of Clinical Infection, Microbiology and Immunology, University of Liverpool Institute of Infection and Global Health , Liverpool, England
[2] Alder Hey Children's Hospital, Department of Infectious Diseases, Eaton Road, Liverpool, L12 2AP
[3] Alder Hey Children's Hospital, Clinical Research Business Unit, Eaton Road, Liverpool, L12 2AP

Micropathology Ltd

Colin G Fink[1,2], Elli Pinnock[1]

[1]Micropathology Ltd Research and Diagnosis
[2]University of Warwick

Newcastle partner

Principle Investigator Marieke Emonts[1,2]

Co-Investigator Rachel Agbeko[1,3]

[1] Institute of Cellular Medicine, Newcastle University, Newcastle upon Tyne, United Kingdom

[2] Paediatric Infectious Diseases and Immunology Department, Newcastle upon Tyne Hospitals Foundation Trust, Great North Children's Hospital, Newcastle upon Tyne, United Kingdom

[3] Paediatric Intensive Care Unit, Newcastle upon Tyne Hospitals Foundation Trust, Great North Children's Hospital, Newcastle upon Tyne, United Kingdom

Gambia partner

Suzanne Anderson: Principal Investigator and West African study oversight:

Fatou Secka: Clinical research fellow and study co-ordinator

Additional Gambia site team (consortium members):

Kalifa Bojang: co-PI

Isatou Sarr: Senior laboratory technician

Miriam Wathuo - local statistician

Ngane Kebbeh: Junior laboratory technician

Gibbi Sey: lead research nurse Medical Research Council Clinic

Momodou Saidykhan: lead research nurse Edward Francis Small Teaching Hospital

Fatoumatta Cole: Data manager

Gilleh Thomas: Data manager

Anna Roca: local collaborator

Akram Zaman - local collaborator

Martin Antonio: Local collaborator

Austrian partner

PI: Werner Zenz[1]
Co-Investigators/Steering committee:
Daniela S. Klobassa[1], Alexander Binder[1], Nina A. Schweintzger[1], Manfred Sagmeister[1]
[1]University Clinic of Paediatrics and Adolescent Medicine, Department of General Paediatrics, Medical University Graz, Austria

Austrian network, participating centres in Austria, Germany, Italy, Serbia, Lithuania, patient recruitment (in alphabetical order):

Hinrich Baumgart[1], Markus Baumgartner[2], Uta Behrends[3], Ariane Biebl[4], Robert Birnbacher[5], Jan-Gerd Blanke[6], Carsten Boelke[7], Kai Breuling[3], Jürgen Brunner[8], Maria Buller[9], Peter Dahlem[10], Beate Dietrich[11], Ernst Eber[12], Johannes Elias[13], Josef Emhofer[2], Rosa Etschmaier[14], Sebastian Farr[15], Ylenia Girtler[16], Irina Grigorow[17], Konrad Heimann[18], Ulrike Ihm[19], Zdenek Jaros[20], Hermann Kalhoff[21], Wilhelm Kaulfersch[22], Christoph Kemen[23], Nina Klocker[24], Bernhard Köster[25], Benno Kohlmaier[26], Eleni Komini[27], Lydia Kramer[3], Antje Neubert[28], Daniel Ortner[29], Lydia Pescollderungg[16], Klaus Pfurtscheller[30], Karl Reiter[31], Goran Ristic[32], Siegfried Rödl[30], Andrea Sellner[26], Astrid Sonnleitner[26], Matthias Sperl[33], Wolfgang Stelzl[34], Holger Till[1], Andreas Trobisch[26], Anne Vierzig[35], Ulrich Vogel[12], Christina Weingarten[36], Stefanie Welke[37], Andreas Wimmer[38], Uwe Wintergerst[39], Daniel Wüller[40], Andrew Zaunschirm[41], Ieva Ziuraite[42], Veslava Žukovskaja[42]

[1]Department of Pediatric and Adolescence Surgery, Division of General Pediatric Surgery, Medical University Graz, Austria
[2]Department of Pediatrics, General Hospital of Steyr, Austria
[3]Department of Pediatrics/Department of Pediatric Surgery, Technische Universität München (TUM), Munich, Germany
[4]Department of Pediatrics, Kepler University Clinic, Medical Faculty of the Johannes Kepler University, Linz, Austria
[5]Department of Pediatrics and Adolesecent Medicine LKH Villach, Austria
[6]Department of Pediatrics and Adolescent Medicine and Neonatology, Hospital Ludmillenstift, Meppen, Germany
[7]Hospital for Children's and Youth Medicine, Oberschwabenklinik, Ravensburg, Germany
[8]Department of Pediatrics, Medical University Innsbruck, Austria
[9]Clinic for Paediatrics and Adolescents Medicine, Sana Hanse-Klinikum Wismar, Germany
[10]Departement of Pediatrics, Medical Center Coburg, Germany
[11]University Medicine Rostock, Department of Pediatrics (UKJ), Rostock, Germany
[12]Department of Pulmonology, Medical University Graz, Austria
[13]Institute for Hygiene and Microbiology, University of Würzburg, Germany
[14]Clinical Institute of Medical and Chemical Laboratory Diagnostics, Medical University Graz, Austria
[15]Department of Pediatric Orthopedics and Adult Foot and Ankle Surgery, Orthopedic Hospital Speising, Vienna, Austria

[16]Department of Paediatrics, Regional Hospital Bolzano, Italy
[17]Department of Pediatrics and Adolescent Medicine, General Hospital Hochsteiermark/Leoben, Austria
[18]Department of Neonatology and Paediatric Intensive Care, Children's University Hospital, RWTH Aachen, Germany
[19]Paediatric Intensive Care Unit, Department of Paediatric Surgery, Donauspital Vienna, Austria
[20]Department of Pediatrics, General Public Hospital, Zwettl, Austria
[21]Pediatric Clinic Dortmund, Germany
[22]Department of Pediatrics and Adolescent Medicine, Klinikum Klagenfurt am Wörthersee, Klagenfurt, Austria
[23]Catholic Children's Hospital Wilhelmstift, Department of Pediatrics, Hamburg, Germany
[24]Department of Pediatrics, Krankenhaus Dornbirn, Austria
[25]Children’s Hospital Luedenscheid, Maerkische Kliniken, Luedenscheid, Germany
[26]Department of General Paediatrics, Medical University Graz, Austria
[27]Department of Paediatrics, Schwarzwald-Baar-Hospital, Villingen-Schwenningen, Germany
[28]Department of Paediatrics and Adolescents Medicine, University Hospital Erlangen, Germany
[29]Department of Pediatrics and Adolescent Medicine, Medical University of Salzburg, Austria
[30]Paediatric Intensive Care Unit, Medical University Graz, Austria
[31]Dr. von Hauner Children's Hospital, Ludwig-Maximilians- Universitaet, Munich, Germany
[32]Mother and Child Health Care Institute of Serbia, Belgrade, Serbia
[33]Department of Pediatric and Adolescence Surgery, Division of Pediatric Orthopedics, Medical University Graz, Austria
[34]Department of Pediatrics, Academic Teaching Hospital, Landeskrankenhaus Feldkirch, Austria
[35]University Children’s Hospital, University of Cologne, Germany
[36]Department of Pediatrics and Adolescent Medicine Wilheminenspital, Vienna, Austria
[37]Department of Pediatric Surgery, Municipal Hospital Karlsruhe, Germany
[38]Hospital of the Sisters of Mercy Ried, Department of Pediatrics and Adolescent Medicine, Ried, Austria
[39]Hospital St. Josef, Braunau, Austria
[40]Christophorus Kliniken Coesfeld Clinic for Pediatrics, Coesfeld, Germany
[41]Department of Paediatrics, University Hospital Krems, Karl Landsteiner University of Health Sciences, Krems, Austria
[42]Children‘s Hospital, Affiliate of Vilnius University Hospital Santariskiu Klinikos, Lithuania
